# Assessment of mid-upper arm circumference for detecting obesity in pregnant women: a cross-sectional study

**DOI:** 10.3389/fgwh.2025.1554068

**Published:** 2025-11-17

**Authors:** Moayad H. Ali, Nadiah AlHabardi, Ishag Adam

**Affiliations:** 1 Faculty of Medicine, University of Medical Sciences and Technology, Kigali, Rwanda; 2 Department of Obstetrics and Gynecology, College of Medicine, Qassim University, Buraydah, Saudi Arabia

**Keywords:** pregnancy, age, mid-upper arm circumference, body mass index, obesity

## Abstract

**Introduction:**

Limited studies have assessed the accuracy of mid-upper arm circumference (MUAC) in diagnosing nutritional status among pregnant women in Sub-Saharan Africa, and none in Rwanda. This study aimed to evaluate the effectiveness of MUAC in detecting obesity among pregnant women at Kacyiru Hospital in Kigali, Rwanda.

**Methods:**

This cross-sectional study was conducted at Kacyiru Hospital, a district hospital in Kigali, Rwanda. Standard procedures were used to measure MUAC, weight, and height, from which body mass index (BMI) was calculated. Receiver operating characteristic (ROC) curves were created to determine cutoff points using Youden's index (YI).

**Results:**

A total of 689 women were enrolled. The median (interquartile range) age and gravidity were 29.0 (26.0–33.0) years and 2 (1–3), respectively. Among the 592 women (85.9%) with gestational ages of ≥20.0 weeks, 5 (0.7%) were underweight and 195 (28.3%) were obese. There was a significant correlation between BMI and MUAC (*r* = 0.78) across all women and within the early (*r* = 0.774) and late pregnancy subgroups. The optimal MUAC cutoff for detecting obesity (BMI ≥ 30.0 kg/m²) was ≥27.5 cm in both early and late pregnancies (YI = 0.58, sensitivity = 0.91, specificity = 0.67), with a high predictive value [area under the receiver operating characteristic curve (AUROCC) = 0.88, 95% confidence interval (CI) = 0.85–0.90]. In early pregnancy, the best MUAC cutoff was ≥29.5 cm (YI = 0.73, sensitivity = 0.92, specificity = 0.80), with a high predictive value (AUROCC = 0.87, 95% CI = 0.77–0.97). In late pregnancy, the best MUAC cutoff was ≥27.5 cm (YI = 0.62, sensitivity = 0.92, specificity = 0.71), with a high predictive value (AUROCC = 0.89, 95% CI = 0.87–0.92).

**Conclusion:**

MUAC is a reliable indicator for detecting obesity in pregnant women. Further research with larger sample sizes and follow-up studies is needed to assess MUAC's ability to detect underweight status and related adverse pregnancy effects.

## Introduction

Recent global reports (1) have highlighted a significant increase in the prevalence of overweight or obesity across all age groups, including in Sub-Saharan countries such as Rwanda (2). This trend extends to pregnant women worldwide (3). Being overweight or obese during pregnancy is associated with numerous adverse maternal and perinatal outcomes, such as gestational diabetes mellitus, gestational hypertension, increased rates of operative delivery, preterm birth, congenital malformations, and postpartum hemorrhage (4).

Preventing obesity during pregnancy and its complications requires effective measures. These measures depend on accurately assessing the extent of the problem, typically achieved through body mass index (BMI) evaluation (5, 6). However, BMI assessment is not without limitations, including the need for technical training and complex calculations. In contrast, mid-upper arm circumference (MUAC) exhibits minimal changes during pregnancy, making it a more reliable indicator of nutritional status during this period (7). Therefore, identifying an optimal alternative to BMI for assessing obesity is crucial (8).

Previous studies have demonstrated a significant positive correlation between MUAC and BMI among adults, including women of reproductive age, in various populations (8–10). However, limited data exist on the correlation between BMI and MUAC during pregnancy, with varying levels of correlation reported (7, 11–13). These studies have suggested different MUAC cutoff points for detecting obesity during pregnancy (7, 11). Moreover, MUAC has been proven effective in accurately detecting not only obesity but also excessive gestational weight gain (14) and undernutrition, along with their associated adverse effects, such as low birth weight (15). Few studies have examined the accuracy of MUAC in diagnosing nutritional status among pregnant women in Sub-Saharan Africa, and none have examined it in Rwanda. Given that anthropometric parameters, including MUAC, can vary across populations, there is an urgent need to establish local cutoff points for use during antenatal care. Therefore, this study aimed to assess MUAC's performance in detecting obesity among pregnant women in Rwanda.

## Methods

### Study area

This cross-sectional study was conducted at Kacyiru Hospital, a district hospital in Kigali, Rwanda, with a specialized maternal and child health unit that provides comprehensive services, including antenatal care, labor and delivery, obstetric and gynecological surgeries, and family planning. The medical staff comprises obstetrics and gynecology consultants, specialists, general practitioners, midwives, and nurses. Approximately 70–90 women attend antenatal consultations daily.

### Study population

The study population included all pregnant women attending antenatal consultations at Kacyiru Hospital.

### Inclusion criteria

The participants included all healthy pregnant women in early and late pregnancies carrying single fetuses and residing in Rwanda, as well as those who provided informed consent before participation.

### Exclusion criteria

The following women were excluded: those with multiple pregnancies; those with chronic diseases, such as HIV, diabetes mellitus, hypertension, and thyroid disease; those who refused to participate; and those with pregnancy complications, such as hyperemesis gravidarum.

### Sampling technique and sample size calculation

Hospital records indicated that 2,078 women attended antenatal consultations three months before the study initiation. Therefore, the required sample size of 689 women was determined by dividing the expected number of women (2,078) by the required sample size (689), yielding a ratio of approximately 3:1. Based on the selection criteria, eligible women were asked to sign an informed consent form. Their age, parity, and the date of their last menstrual period were recorded. Gestational age was calculated from the last menstrual period and confirmed by early-pregnancy ultrasound. MUAC was measured with a non-stretchable tape measure, with the midpoint between the acromion and olecranon processes used as the measurement point. Height was measured using a Seca 786 combinable stadiometer with a weighing scale (Hammer Steindamm 3–25, 22089 Hamburg, Deutschland, Germany). The participants stood barefoot, ensuring their heels, buttocks, and shoulder blades touched the back plate, with their heads positioned in the Frankfurt horizontal plane. Weight was measured using the same Seca 786 scale after the participants removed heavy clothing and shoes. Each measurement was taken twice, and the average was calculated; a third measurement was taken if there was any discrepancy between the initial measures. All instruments were calibrated daily. BMI was calculated using the standard formula: weight (kg)/height (m)^2^. The <18.5 kg/m^2^ and ≥30.0 kg/m^2^ BMI cutoff points were used to identify underweight and obese women, respectively.

### Sample size calculation

Using an online sample size calculator (16) and based on a previous publication (17), we assumed that 30% of pregnant women in Kigali, Rwanda, would be obese. We further assumed that MUAC would have a sensitivity of 95% in detecting obesity among these pregnant women. A sample size of 689 women was used to achieve a 95% confidence interval with a precision of 0.05.

### Statistics

The data were analyzed using SPSS for Windows, version 22.0 (IBM Corp., NY, USA). The age, gravidity, BMI, and MUAC of the participants were assessed for normality using the Shapiro–Wilk test and found to be non-normally distributed. Therefore, these data were expressed as median (interquartile range, IQR) and compared between women at early and late gestational ages using the non-parametric Mann–Whitney U test. A receiver operating characteristic (ROC) analysis was performed to assess the sensitivity, specificity, and optimal cutoffs of MUAC for detecting obesity in pregnant women. The ROC parameters were determined based on the highest Youden's index (YI), calculated as YI = sensitivity + specificity−1.

## Results

**Figure 1:**
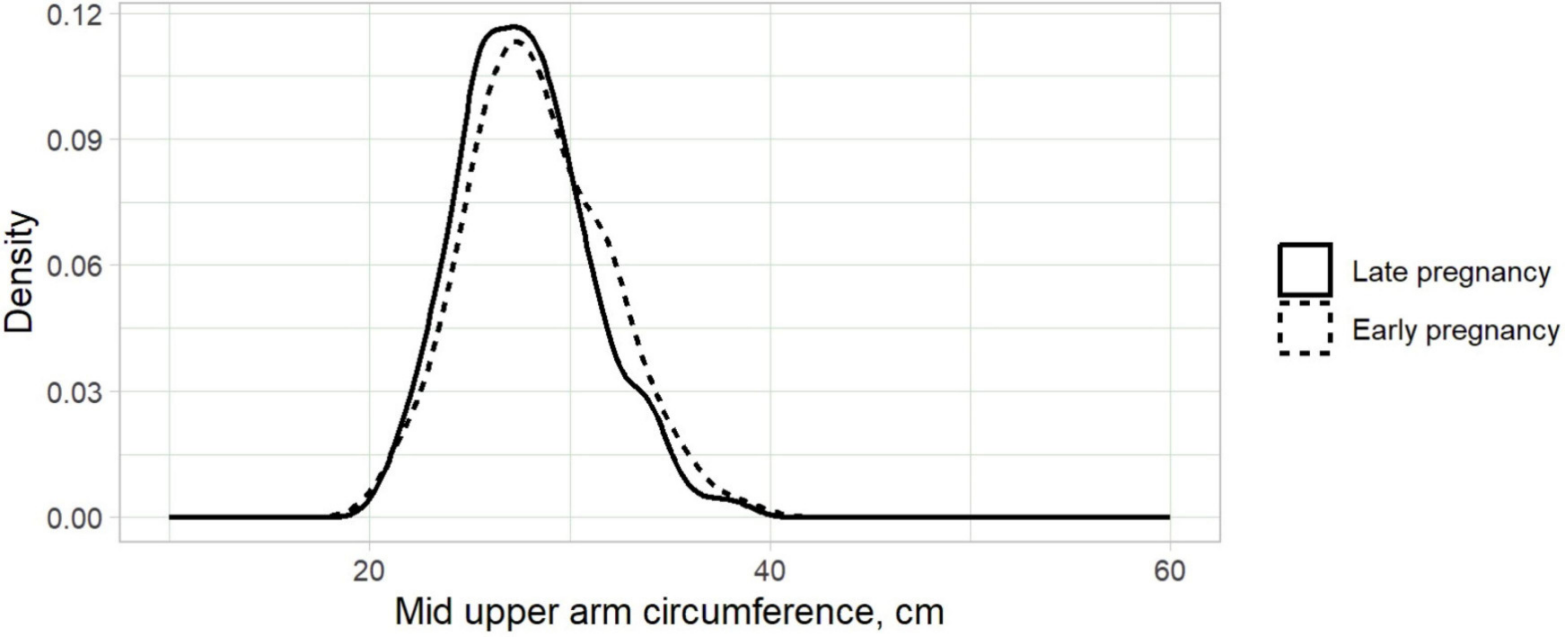
Kernel density plot of mid-upper arm circumference for pregnant women in Kigali, Rwanda, 2024.

**Figure 2:**
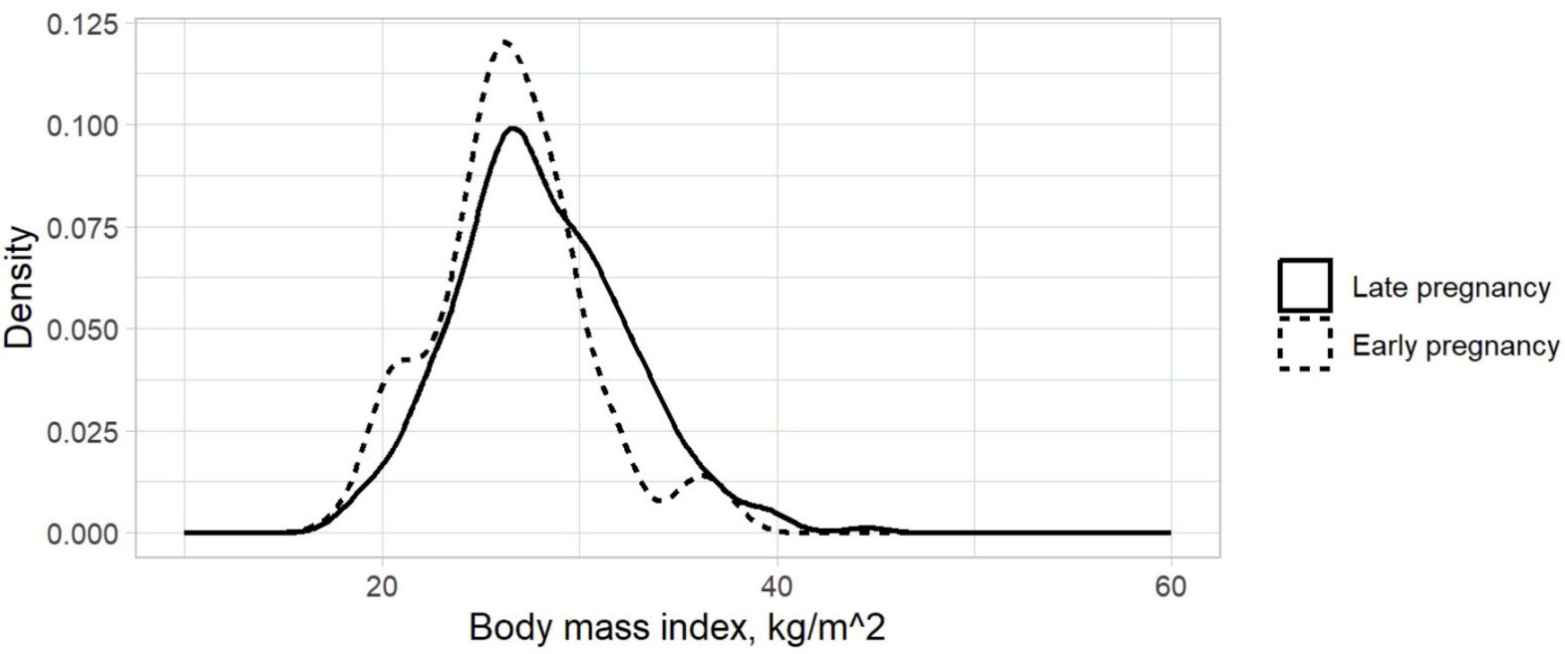
Kernel density plot of body mass index for pregnant women in Kigali, Rwanda, 2024.

A total of 689 women were enrolled in the study. The median (IQR) age and gravidity of the participants were 29.0 (26.0–33.0) years and 2 (1–3), respectively. MUACs were 22.2–39.0 (median: 27.5) cm (Figure 1). The gestational ages were 8.0–40.0 (median: 33.0) weeks, and 592 (85.9%) of the women were at gestational ages ≥20.0 weeks. BMIs were 18.0–44.6 (median: 27.3 kg/m²) (Figure 2). Only 5 (0.7%) women were underweight, while 195 (28.3%) were classified as obese.

**Figure 3:**
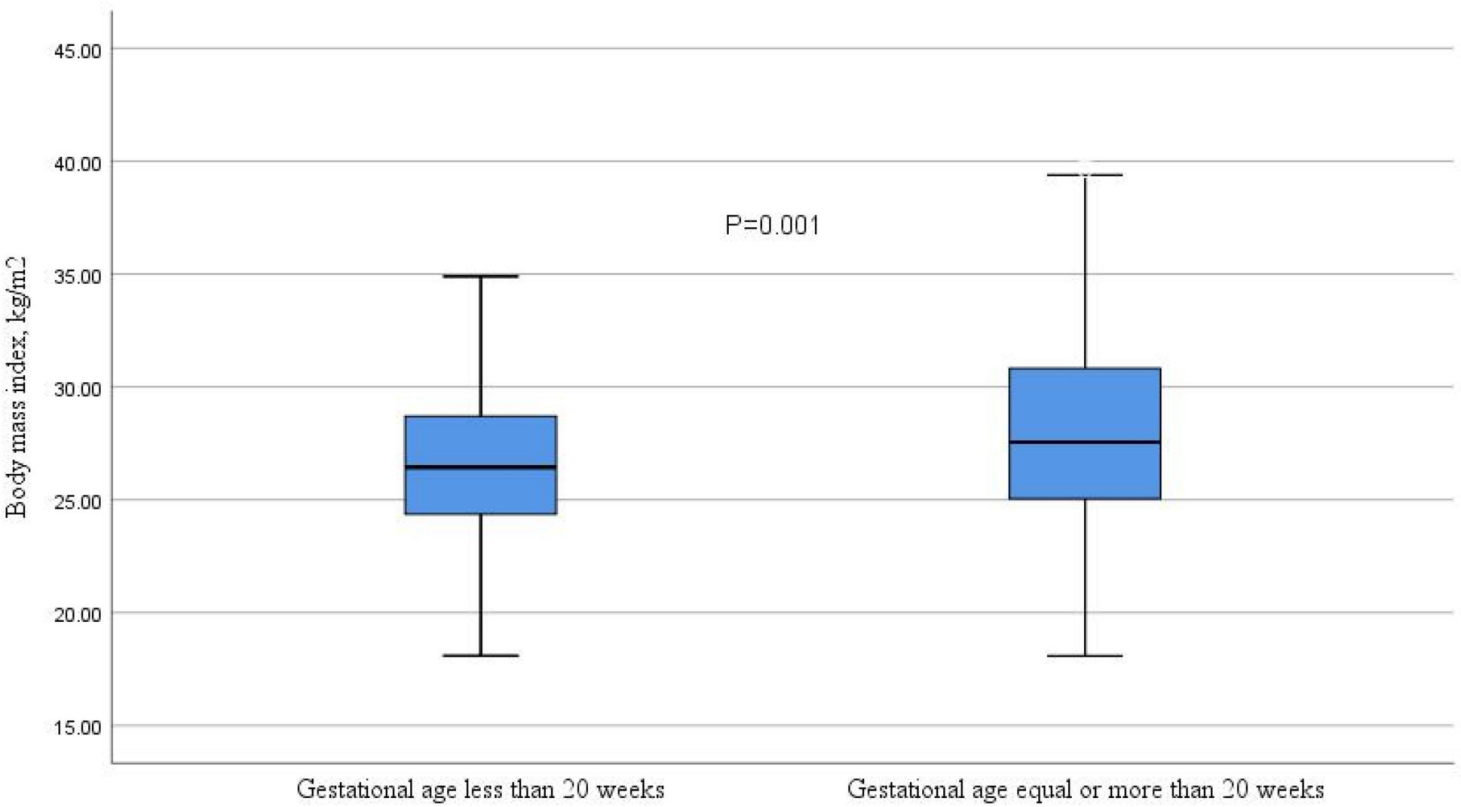
Comparison of body mass index in early and late pregnancies in Kigali, Rwanda, 2024.

**Figure 4:**
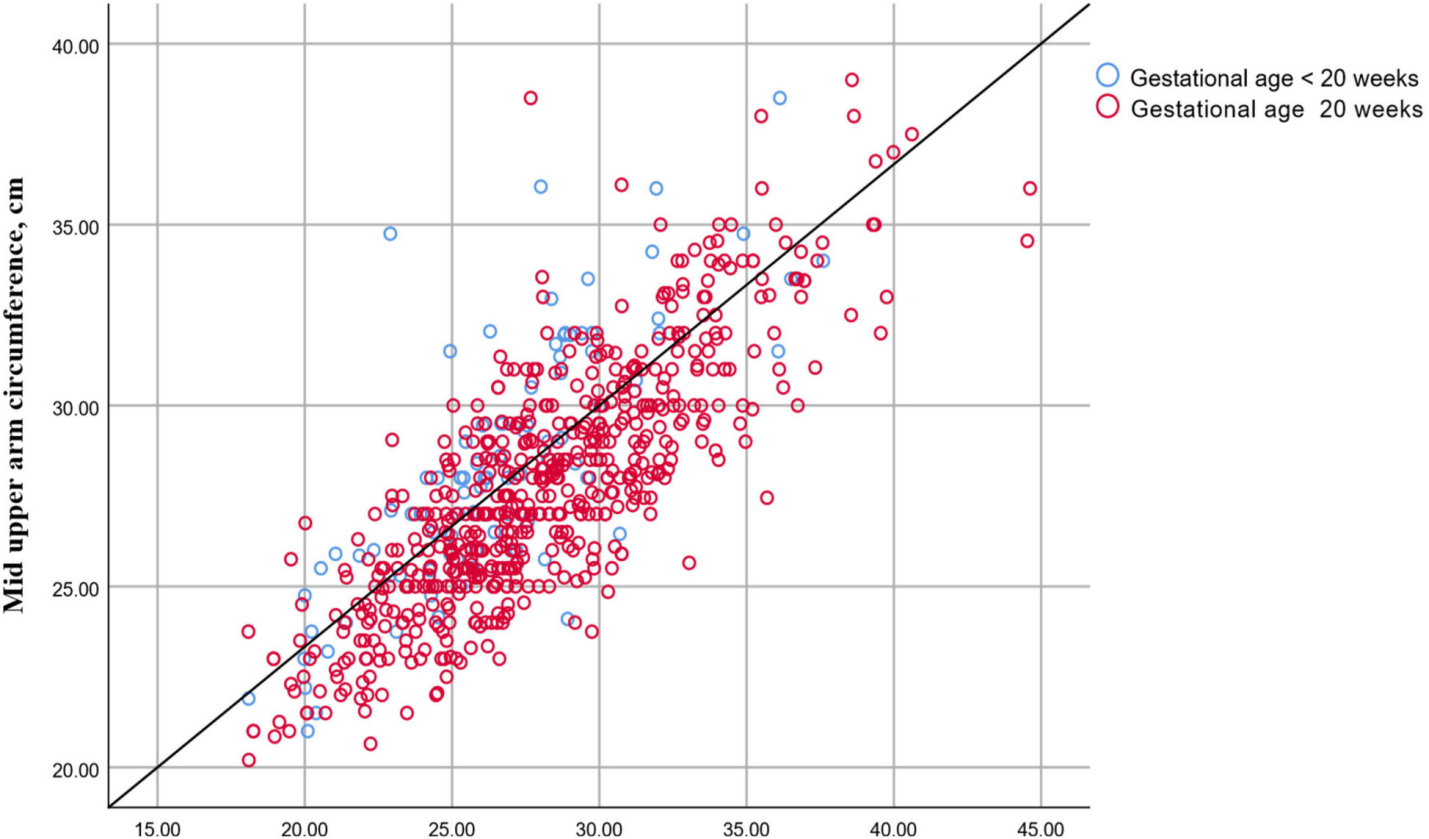
Correlation between body mass index and mid-upper arm circumference in pregnant women in Kigali, Rwanda, 2024.

The median (IQR) BMI was significantly higher in women in late pregnancy (Figure 3). However, age, gravidity, and MUAC did not differ significantly between women in early and late pregnancies (Table 1). BMI and gestational age were positively correlated in all women (*r* = 0.188, *P* < 0.001), particularly in those in late pregnancy (*r* = 0.152, *P* < 0.001). In contrast, no significant correlation was found between BMI and gestational age among women in early pregnancy (*r* = 0.067, *P* = 0.517). A significant correlation was observed between BMI and MUAC in all women (*r* = 0.78, *P* < 0.001), as well as in both early pregnancy (*r* = 0.774, *P* < 0.001) and late pregnancy (*r* = 0.806, *P* < 0.001) (Figure 4).

**Table 1:** Comparison of age, gravidity, body mass index, and mid-upper arm circumference between women in early and late gestational ages at Kigali, Rwanda, 2024.

Variables	Women in early pregnancy (97)	Women in late pregnancy (592)	*P* value
Age, years	30.0 (26.0–34.0)	29.0 (26.0–33.0)	0.545
Gravidity	2 (1–3)	2 (1–3)	0.272
Body mass index, kg/m^2^	26.4 (24.3–28.6)	27.5 (25.0–30.8)	0.001
Mid-upper arm circumference, cm	28.0 (25.9–30.9)	27.5 (24.4–29.8)	0.108

**Figure 5:**
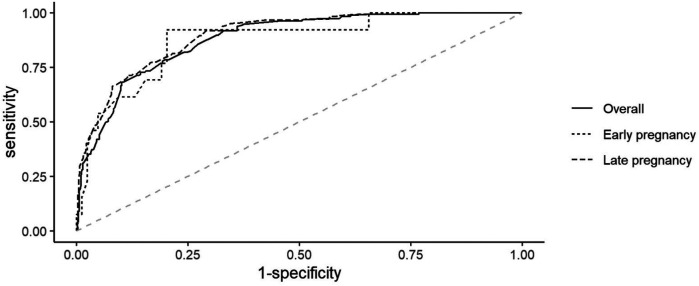
Receiver operating curve of mid-upper arm circumference for diagnosing obesity in pregnant women in Kigali, Rwanda, 2024.

The optimal MUAC cutoff for detecting obesity (BMI ≥ 30.0 kg/m^2^) was ≥ 27.5 cm in both early and late pregnancies [YI = 0.58, sensitivity = 0.91, specificity = 0.67], with a high predictive value [area under the receiver operating characteristic curve (AUROCC) = 0.88, 95% confidence interval (CI) = 0.85–0.90]. In early pregnancy, the best MUAC cutoff was ≥29.5 cm (YI = 0.73, sensitivity = 0.92, specificity = 0.80), with a high predictive value (AUROCC = 0.87, 95% CI = 0.77–0.97). In late pregnancy, the best MUAC cutoff was ≥27.5 cm (YI = 0.62, sensitivity = 0.92, specificity = 0.71), with a high predictive value (AUROCC = 0.89, 95% CI = 0.87–0.92) (Table 2, Figure 5).

**Table 2:** Performance of mid-upper arm circumference for detecting obesity in pregnant women in Kigali, Rwanda, 2024.

Variables	Total pregnant women	Women in early pregnancy (*n* = 97)	Women in late pregnancy (*n* = 592)
Area under the receiver operating characteristic curve	0.88	0.87	0.89
Cutoff, cm	≥27.5	≥29.5	≥27.5
Sensitivity	0.91	0.92	0.92
Specificity	0.67	0.80	0.71
Youden's index	0.58	0.73	0.62

## Discussion

MUAC and BMI were significantly correlated among pregnant women (*r* = 0.78). This correlation was consistently observed in early (*r* = 0.774) and late (*r* = 0.806) pregnancies. These findings are consistent with previous studies that reported strong correlations between MUAC and BMI among pregnant women (7, 11–13). For instance, a study in Sudan found that MUAC and BMI were positively correlated among 688 pregnant women, with correlations of 0.734 in early pregnancy (<20 weeks) and 0.703 in late pregnancy (≥20 weeks) (12). Similarly, Fakier et al. reported that MUAC and BMI were strongly correlated in South Africa (*r* = 0.93) in early pregnancy (<20 weeks) and (*r* = 0.92) in late pregnancy (≥20 weeks) (11). Miele et al., in a large Brazilian study involving 1,165 pregnant women, reported significant correlations (*r* = 0.872) in the first trimester, (*r* = 0.870) in the second trimester, and (*r* = 0.831) in the third trimester (7). Cooley et al., in a study across London and Dublin with 2,912 pregnant women, also found that MUAC and BMI were positively correlated (*r* = 0.836) (13). Previous studies among non-pregnant women have shown that MUAC and BMI are positively correlated. For example, studies in Sudan (*r* = 0.639) (8), Iran (*r* = 0.91) (18), and India (*r* = 0.86) (19).

We identified a MUAC cutoff point of ≥27.5 cm for detecting obesity, demonstrating good predictive value (AUROCC = 0.88, YI = 0.58, sensitivity = 0.91, specificity = 0.67). A slightly higher MUAC cutoff of 28.0 cm was reported in Sudanese women in early pregnancy, albeit with somewhat lower accuracy (YI = 0.61, sensitivity = 76%, specificity = 86%) (12). For late pregnancy in the same study, the MUAC cutoff was 29 cm (YI = 0.67, sensitivity = 80.0%, specificity = 87.0%) (12). Comparatively, our identified MUAC cutoff point for obesity (≥27.5 cm) was lower than that reported in South Africa (30.5 cm) (11) and Brazil (30.15 cm for early pregnancy and 30.6 cm for late pregnancy) (7). These differences in MUAC cutoffs and their respective sensitivities and specificities may stem from variations in nutritional status, sociodemographic characteristics, genetics, and methodological differences. To determine the cutoff points, we used YI, which was not used in most previous studies. Although BMI differed significantly between early and late pregnancies, MUAC did not differ significantly between these stages in our study. Previous research has also noted variations in BMI across pregnancy stages (7), highlighting the potential advantage of using MUAC over BMI for assessing nutritional status during pregnancy.

Given the variability in BMI across gestational ages, assessing weight gain during pregnancy might offer a better indicator of nutritional status. Nonetheless, MUAC reliably assesses the rate of gestational weight gain as well (14).

### Strengths and limitations

Strengths: This study addresses an important gap in maternal health research in Rwanda, where limited data exist on the use of MUAC as a screening tool for obesity in pregnancy. The sample size (*n* = 689) is large and provides sufficient statistical power to support the findings. Methods are clearly described, including calibration of instruments and standardized measurement protocols, which strengthens reliability. Use of ROC analysis and Youden's index to determine optimal MUAC cutoff points is statistically robust and appropriate. Findings are relevant to antenatal care in low-resource settings, where BMI measurements may be more difficult to obtain.

Limitations: Very few underweight women were included (0.7%, *n* = 5), which prevents meaningful conclusions on MUAC's ability to detect undernutrition. Pre-pregnancy BMI and gestational weight gain were not assessed, limiting understanding of MUAC's predictive role throughout pregnancy. The single-center, hospital-based setting in Kigali restricts generalizability to the wider Rwandan or Sub-Saharan African population. The cross-sectional design precludes conclusions on causality or prediction of maternal/neonatal outcomes. Analyzing factors such as education, income, and dietary habits could provide insights into the underlying causes of obesity and undernutrition. Moreover, we did not follow these women until delivery and assessed their maternal and perinatal outcomes, whether associated with BMI or MUAC. Investigating the relationship between MUAC and specific pregnancy complications (e.g., gestational diabetes, preterm birth, low birth weight) would enhance the clinical relevance of the study.

## Conclusion

MUAC demonstrates good reliability in detecting obesity among pregnant women. Future research with larger sample sizes and longitudinal follow-up is warranted to further investigate MUAC's utility in detecting underweight and assessing pregnancy-related adverse effects.

## Data Availability

The original contributions presented in the study are included in the article/Supplementary Material, further inquiries can be directed to the corresponding author.
